# Differences in coagulofibrinolytic changes between post‐cardiac arrest syndrome of cardiac causes and hypoxic insults: a pilot study

**DOI:** 10.1002/ams2.270

**Published:** 2017-03-27

**Authors:** Takeshi Wada, Satoshi Gando, Asumi Mizugaki, Akira Kodate, Yoshihiro Sadamoto, Hiromoto Murakami, Kunihiko Maekawa, Kenichi Katabami, Yuichi Ono, Mineji Hayakawa, Atsushi Sawamura, Subrina Jesmin, Masahiro Ieko

**Affiliations:** ^1^ Division of Acute and Critical Care Medicine Department of Anesthesiology and Critical Care Medicine Hokkaido University Graduate School of Medicine Sapporo Japan; ^2^ Deparment of Emergency and Critical Care Medicine Faculty of Medicine University of Tsukuba Tsukuba Japan; ^3^ Department of Internal Medicine School of density Health Sciences University of Hokkaido Tobetsu Japan

Dear Editor,

Post‐cardiac arrest syndrome (PCAS) is often involved in coagulofibrinolytic disorder, which occurs as a result of systemic ischemia and reperfusion.^1^ We are under the clinical impression that PCAS patients who experience cardiac arrest due to hypoxia are inclined to suffer from severe coagulopathy and that their condition is associated with a worse prognosis in comparison to patients in whom cardiac arrest occurs in association with a cardiogenic event.

The present study is a subgroup analysis of our previous study.^1^ Thirteen patients with PCAS caused by cardiogenic cardiac arrest (the cardiogenic group) and 13 patients with PCAS caused by hypoxia‐related cardiac arrest (the hypoxia group) were enrolled in the present study. Soluble fibrin (SF) and plasmin‐α2 plasmin inhibitor complex (PPIC), which are markers of thrombin activation and plasmin activation, respectively, were measured.

Figure 1 shows the serial changes in the plasma levels of SF and PPIC. On day 1, the levels of SF in the hypoxia group were significantly higher than that in the cardiogenic group. Moreover, the levels of PPIC in the hypoxia group were significantly elevated in comparison to the cardiogenic group on day 1. When a good outcome was defined as cerebral performance categories 1 or 2, and a poor outcome was defined as cerebral performance categories 3–5, the cardiogenic group showed a more favorable outcome than the hypoxia group (good / poor: 4/9 versus 0/13, respectively; *P* = 0.003). The intervals between the receipt of the emergency call and the return of spontaneous circulation in the hypoxia and cardiogenic groups did not differ to a statistically significant extent (cardiogenic versus hypoxia, 38.9 ± 4.2 min versus 31.1 ± 2.3 min, *P* = 0.111).

**Figure 1:**
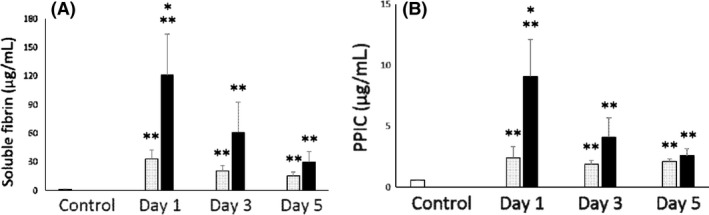
Bar graphs showing serial changes in soluble fibrin (A) and plasmin‐α2 plasmin inhibitor complex (PPIC) (B) in patients with post‐cardiac arrest syndrome (PCAS). Black bars, PCAS caused by hypoxia‐related cardiac arrest (hypoxia group); gray bars, PCAS caused by cardiogenic cardiac arrest (cardiogenic group); white bars, control subjects. *P < 0.05 cardiogenic group versus hypoxia group; **P < 0.05 versus control subjects.

Our previous study suggested that the SF levels of PCAS patients with disseminated intravascular coagulation (DIC) were significantly higher than those in patients without DIC.^1^ Systemic hypoxia, ischemia, and reperfusion cause endothelial damage with a consequent increase in tissue factor activity, which forms a complex with factor VII/VIIa, and the complex results in the generation of thrombin.^2^ Moreover, anoxia and endothelial injury lead to hyperfibrinolysis.^3^ These findings indicate that the hypoxia‐induced endothelial injury of the hypoxic PCAS patients was more serious. This could result in more severe coagulopathy with hyperfibrinolysis, leading to a poorer outcome. This may be due to the differences in the pre‐cardiac arrest conditions of patients with cardiogenic and hypoxic PCAS. Hypoxic cardiac arrest is affected by both hypoxia due to circulatory arrest and pre‐cardiac arrest hypoxia, followed by more severe endothelial damage and coagulofibrinolytic changes. The results of the present study are also supported by the previous study, which showed that patients with cardiogenic PCAS had a better chance of surviving until discharge and a more favorable long‐term outcome than patients with a non‐cardiac etiology, including a large number of patients with hypoxic insults, especially if they had a lower International Society on Thrombosis and Haemostasis DIC score.^4^

We believe that our results will be helpful for the further investigations on the pathophysiology of PCAS and for the development of therapeutic strategies.

## Funding Information

Grant‐in Aid for Young Scientists (B) from the Ministry of Education, Science, Sports and Culture of Japan, 2011‐237920912013‐25861736

## Conflict of Interest

All of the study participants provided their informed consent, and the study design was approved by an ethics review board. Takeshi Wada received honoraria from Asahi Kasei Pharma, manuscript fees from Herusu Syuppan and Sougou Igakusya, and research funding from Senshin Medical Research Funding. Satoshi Gando received honoraria from Asahi Kasei Pharma and Nihon Seiyaku, manuscript fees from Asahi Kasei Pharma, fees for promotional materials from Asahi Kasei Pharma, Nihon Seiyaku, and Japan Blood Products Organization, and research funding from the Ministry of Health, Labor and Welfare of Japan. Mineji Hayakawa received honoraria and research funding from Asahi Kasei Pharma Corporation. The other authors have no conflict of interest.
